# Commentary: The effects of complex training on performance variables in basketball players: a systematic review and meta-analysis

**DOI:** 10.3389/fspor.2025.1747190

**Published:** 2026-01-02

**Authors:** Rohit Kumar Thapa, João Bruno, Exal Garcia-Carrillo

**Affiliations:** 1 Symbiosis School of Sports Sciences, Symbiosis International (Deemed University), Pune, India; 2 CIPER, Faculty of Sport Sciences and Physical Education, University of Coimbra, Coimbra, Portugal; 3 Portugese Air Force, Lisbon, Portugal; 4 Department of Physical Activity Sciences, Faculty of Education Sciences, Universidad Católica del Maule, Talca, Chile; 5 Department of Physical Activity Sciences, Universidad de Los Lagos, Osorno, Chile

**Keywords:** athletic performance, muscle strength, plyometric exercise, post-activation potentiation, resistance training

A Commentary on The effects of complex training on performance variables in basketball players: a systematic review and meta-analysis By Kambitta Valappil IN, Parpa K, Govindasamy K, Katanic B, Clark CCT, Elayaraja M, Karmakar D, Băltean AI, Roxana Forț P and Geantă VA (2025). Front. Sports Act. Living. 7:2025. doi: 10.3389/fspor.2025.1669334

## 1 Introduction

We read with interest the systematic review and meta-analysis paper by Kambitta Valappil et al. (1) that investigated the effects of complex training on the physical performance of basketball players. While we commend the authors for their valuable effort in aggregating the available studies that examined the effects of complex training on the physical performance of basketball athletes (1), there are some aspects of the paper that merit further consideration and discussion. Therefore, this commentary aims to highlight some key considerations in the paper that may be useful for readers when interpreting the findings.

## 2 Definition of complex training

While the terms ‘complex training’ and ‘contrast training’ have been used interchangeably in the sport and exercise science literature over the last two decades, Cormier et al. (2) clarified and proposed the use of ‘complex training’ as a broad umbrella term in their recent paper. According to their definition, complex training is an exercise format that involves performing heavy resistance exercises (higher load, lower velocity) and ballistic exercises (lower load, higher velocity) within a single session. Furthermore, the heavy resistance exercise and ballistic exercise can be performed in four different sequences, namely, complex-ascending training (all ballistic exercise performed first, followed by completion of heavy resistance exercise at the end), complex-descending training (all heavy resistance exercise performed first, followed by ballistic exercises at the end), complex-contrast training (heavy resistance exercise and ballistic exercise paired and performed in a set-by-set fashion), and French contrast training (heavy resistance exercise, unloaded ballistic exercise, loaded ballistic exercise, assisted ballistic exercise performed in a sequence).

However, Kambitta Valappil et al. (1) provided only a brief and narrow definition of complex training, primarily framed around the acute post-activation potentiation mechanism and alteration of heavy resistance and plyometric exercises. This brevity may unintentionally lead readers to assume that all formats involving resistance and ballistic exercises within the same session operate through the same underlying rationale, which is not necessarily the case. A more precise conceptualization of complex training in the introduction, distinguishing complex-contrast formats from other within-session combinations of resistance and ballistic exercises (2), would have provided clarity and improved interpretability.

We provide some excerpt from the study. For example, in the introduction, the authors defined complex training as:

“For this review, CT specifically refers to this strength plyometric pairing and its physiological basis. CT is prefaced on Post-Activation Potentiation (PAP), a transient increase in muscle force after intense contraction”, and “CT protocols typically alternate high-load weight training (75%–90% 1RM, 2–12 repetitions) with maximum intensity plyometrics (5–15 repetitions) within the same workout session.”

Similarly, in the discussion, the authors supported the improvement in performance with this rationale and wrote:

“The study results indicate that CT enhances vertical jump performance, a benefit that can be partly explained by the PAP theory.”

Based on the introduction and discussion sections, the mechanistic rationale provided is that of complex-contrast training, which involves alternating heavy resistance and ballistic exercises in a set-by-set fashion within a session (3). However, the inclusion criteria (i.e., all combinations of resistance and ballistic exercise performed in a single session) (Table 1) and the definition of complex training presented in the study are inconsistent, as not all formats of complex training are suggested to induce post-activation performance enhancement. For example, in a complex-ascending format, ballistic exercises are performed first, followed by a resistance exercise at the end of the session, which does not align with the post-activation performance enhancement rationale of heavy-load potentiating lighter-load activity.

Additionally, it is worth noting that post-activation potentiation and post-activation performance enhancement are two distinct theoretical concepts, which have been recently discussed by researchers (4, 5). Both mechanisms differ in timing and physiological basis, and therefore should not be used interchangeably. For example, post-activation potentiation is suggested to invoke at ∼28 s (due to myosin regulatory light chain phosphorylation), whereas improvements at 3–12 min duration after a conditioning activity is suggested to be induced due to post-activation performance enhancement (potentially due to increase in muscle temperature, muscle and muscle fiber water content, muscle activation) (3, 4).

## 3 Reference does not support some key statements

In the introduction section, we were excited to read some interesting statements about the findings of previous studies on complex training. However, the cited reference studies do not support the statement made. For example:

“CT was reported to positively affect sprint abilities, both acutely and chronically. Whilst agility, CoD speed, muscular strength, isometric force, and explosive power, also responded positively, often surpassing traditional weight training (26, 27). Some studies also reported positive effects on aerobic and cardiorespiratory endurance (27).

The reference number 26 is a narrative review paper by Cormie et al. (6), in which the authors hypothesized that a combined training approach may produce higher adaptation compared to isolated training, but does not reflect the findings of an empirical or meta-analytical study. While reference number 27 is a meta-analysis paper on plyometric jump training adaptations, which did not analyze complex training (7). Therefore, the statement that complex training is more effective than single-mode training in improving agility, change-of-direction speed, muscular strength, isometric force, explosive power, and aerobic and cardiorespiratory endurance is not supported by appropriate references.

Another example:

“Despite positive findings, limitations and inconsistencies exist. Indeed, some studies suggest traditional training might be more effective in specific scenarios (18).”

The statement suggests that some studies have shown traditional training to be more effective than complex training. However, reference number 18 is a meta-analysis that analyzed the factors modulating post-activation performance enhancement of jump, sprint, and upper-body ballistic performance (8).

Several other statements in the paper are incorrectly cited, likely due to unintentional oversight. However, due to word count limitations, we have presented only two cases. Of note, accurate citation in the introduction section is crucial, as it frames the current state of knowledge and justifies the research question. Moreover, incorrect references in other sections of the paper (e.g., discussion) can lead to misunderstandings about established evidence and theoretical underpinnings, particularly in sports and exercise science, and may result in the dissemination of incorrect information to readers (9, 10).

## 4 Inclusion and exclusion criteria

The inclusion criteria (PICOS; Table 1) defined the comparator as an active control participating in any form of training, including standard training, traditional resistance training, plyometric training, or other exercise modalities specifically designed for performance enhancement. However, this inclusion criterion has led to the selection of heterogeneous studies. For example, the comparators in the included studies were plyometric training (11), resistance training (12), optimum power load training (13), while three studies used controlled conditions (14–16). The inclusion of varied comparator groups affects the meta-analysis results (e.g., the alternative active training group may have independent ergogenic effects of varying magnitudes), as it increases the between-study heterogeneity (e.g., *I*^2^ = 86%–91% reported in the meta-analysis) and reduces the precision of pooled effect estimates. We believe this to be an important limitation of the study that readers should consider.

Additionally, we observed inconsistencies between the eligibility criteria reported in the article text and those indicated in Table 1. For example, while the text indicates that comparators may include “standard training, traditional resistance training, plyometric training, or other exercise modalities”, Table 1 includes “other interventions” as an exclusion criterion, generating ambiguity regarding the eligible comparators. Likewise, the table excludes “controlled studies,” a designation that technically would include randomized controlled trials, contradicting its previously declared inclusion. The aforementioned discrepancies reduce the clarity and reproducibility of the selection process. Additionally, the detailed search strategy for the Scopus database (Table 2) contains some typographical errors that may yield erroneous search results. For example, “photoactivation potentiation”, “perfoarmance”, “change off direction”, and “1arm” are likely some unintentional typographical errors; however, it may reduce the reproducibility of the search, and therefore this should be considered in future studies.

Furthermore, we found that one ineligible study was included in the meta-analysis due to a screening error, potentially during the study selection process. For one of the included studies (17), Kambitta Valappil et al. (1) stated (in Table 3) that the control group performed compound training [i.e., resistance exercise and plyometric exercise performed on separate days (18)], however, the study compared two different formats of complex training. The study actually reversed the sequence of the exercises in the comparator group (i.e., ballistic exercise performed first, followed by heavy resistance exercise) within the same session, with similar volume and intensity (17). This error may have occurred because the original authors of the study referred to it as compound training (17). However, as per the inclusion criteria framed by the authors (1), since both the intervention groups in the study conducted heavy resistance exercise and ballistic exercise within the same session, they should be categorized as complex training (17), and should have been excluded from the meta-analysis.

## 5 Methodological concerns in the meta-analysis

Firstly, the study by Biel et al. (17) should be excluded from the meta-analysis. Secondly, several studies included multiple intervention and control groups [e.g., (14)], but there is no indication that proper adjustments (e.g., dividing the shared control group sample) were made as per Cochrane guidelines (19). This introduces unit-of-analysis errors (due to double-counting of groups), inflating the weight of certain studies and biasing pooled estimates (20), which in turn provide erroneous results (21). For example, Figures 3, 4 of Kambitta Valappil et al. (1) present the included studies' weight for change of direction speed and vertical jump, respectively. In Figure 1, two different change-of-direction scores were included from Wang. et al. (12). However, the number of participants was not normalized for the number of outcomes selected from the same study (22). The weightage of Wang et al. (12) in the meta-analysis is 48.3% with an overestimated sample size of 64. Similarly, for the vertical jump meta-analysis, the pooled sample size considered is 510; however, the actual sample size to be used in the meta-analysis, based on the included studies, should not exceed 229. The weightage of Hassan et al. (16), Latorre Román et al. (14), and Santos & Janeira (15) has been overestimated in the meta-analysis, as multiple outcomes were selected without normalization of the sample size (23). Additionally, although confidence intervals were presented, prediction intervals (PIs) were omitted. In random-effects meta-analyses, especially with substantial heterogeneity (*I*² = 86%–91% in this case), PIs are essential as they indicate the range within which the true effect of future studies is likely to fall. Their absence compromises the practical applicability of the results, as future effects could plausibly be null or even adverse (24). These statistical errors reduce the precision or interpretability of the pooled estimates.

Furthermore, including countermovement jump, drop jump, and squat jump in the same meta-analysis without a subgroup analysis may not be an optimal choice, considering the distinct neuromuscular ability of each jump type. For example, countermovement jump and drop jump assess the slow and fast stretch-shortening cycle, respectively (25, 26). While the squat jump does not involve a stretch-shortening cycle and assesses concentric-only muscle power (27). This methodological error may lead to inaccurate or misleading conclusions, as including countermovement jump, drop jump, and squat jump without appropriate sub-group analysis fails to account for the distinct neuromuscular mechanisms each jump assesses. This oversight risks conflating different neuromuscular characteristics, thereby obscuring specific effects and potentially leading to erroneous practical recommendations.

Therefore, to help illustrate how methodological decisions may influence effect estimates, we conducted a sensitivity check by performing revised meta-analyses based on the data extracted from the included studies using the DerSimonian and Laird random-effects model. A revised meta-analysis was not conducted for the drop jump, as only two studies (14, 15) provided data.

Three studies provided data on the change of direction speed, involving 108 participants (55 in the complex training group and 53 in the control group). Complex training significantly improved the change of direction performance compared to the control group [Effect size (ES) = −0.75; 95% confidence interval [CI] = −1.15 to −0.35; 95% PI = −8.57 to 6.98; p < 0.001; *I*^2^ = 57.2%].

Five studies provided data for countermovement jump, including 158 participants (83 in the complex training group and 75 in the control group). Complex training significantly improved the countermovement jump performance compared to the control group (ES = 0.58; 95% CI = 0.21 to 0.95; 95% PI = −0.29 to 1.42; *p* = 0.001; *I^2^* = 21.9%).

Three studies provided data for squat jump, including 119 participants (69 in the complex training group and 50 in the control group). No significant difference was observed between the complex training group and the control group (ES = 1.09; 95% CI = −0.05 to 2.23; 95% PI = −3.89 to 5.99; *p* = 0.060; *I*^2^ = 84.7%). After a sensitivity analysis, excluding the data outlier data, similar results were found (ES = 0.46; 95% CI = −0.19 to 1.11; 95% PI = −6.13 to 7.02; *p* = 0.165; *I*^2^ = 53.6%).

## 6 Conclusion

In this commentary on the systematic review and meta-analysis by Kambitta Valappil et al. (1), we respectfully highlight several concerns that warrant consideration by readers for a balanced interpretation of the findings. Furthermore, we also conducted a sensitivity check by performing revised meta-analyses to support the analyses presented by the authors. Nonetheless, we appreciate reading the author's work and commend their efforts put into conducting the meta-analysis. We believe this commentary will provide the readers with a well-balanced critique and complements the existing study.
